# Large language models for closed-library multi-document query, test generation, and evaluation

**DOI:** 10.3389/frai.2025.1592013

**Published:** 2025-08-06

**Authors:** Claire Randolph, Adam Michaleas, Darrell O. Ricke

**Affiliations:** ^1^Department of the Air Force, Artificial Intelligence Accelerator, Cambridge, MA, United States; ^2^AI Technology, MIT Lincoln Laboratory, Lexington, MA, United States

**Keywords:** large language models, LLM, retrieval-augmented generation, RAG, LangChain

## Abstract

**Introduction:**

Learning complex, detailed, and evolving knowledge is a challenge in multiple technical professions. Relevant source knowledge is contained within many large documents and information sources with frequent updates to these documents. Knowledge tests need to be generated on new material and existing tests revised, tracking knowledge base updates. Large Language Models (LLMs) provide a framework for artificial intelligence-assisted knowledge acquisition and continued learning. Retrieval-Augmented Generation (RAG) provides a framework to leverage available, trained LLMs combined with technical area-specific knowledge bases.

**Methods:**

Herein, two methods are introduced (DaaDy: document as a dictionary and SQAD: structured question answer dictionary), which together enable effective implementation of LLM-RAG question-answering on large documents. Additionally, the AI for knowledge intensive tasks (AIKIT) solution is presented for working with numerous documents for training and continuing education. AIKIT is provided as a containerized open source solution that deploys on standalone, high performance, and cloud systems. AIKIT includes LLM, RAG, vector stores, relational database, and a Ruby on Rails web interface.

**Results:**

Coverage of source documents by LLM-RAG generated questions decreases as the length of documents increase. Segmenting source documents improve coverage of generated questions. The AIKIT solution enabled easy use of multiple LLM models with multimodal RAG source documents; AIKIT retains LLM-RAG responses for queries against one or multiple LLM models.

**Discussion:**

AIKIT provides an easy-to-use set of tools to enable users to work with complex information using LLM-RAG capabilities. AIKIT enables easy use of multiple LLM models with retention of LLM-RAG responses.

## 1 Introduction

Some highly technical professions require learning and retention of complex, detailed, and evolving knowledge from multiple relevant documents and information sources. Adding more complexity, these documents are updated with new and changing information on a frequent basis, which makes keeping up-to-date on the most current information a challenging task for these highly technical professionals. In professions with a specified instructor corps, generating and maintaining instructional material on such a dynamic and vast corpus can be overwhelming and time-consuming for instructors. Knowledge tests can assist learners in encoding and retaining new knowledge, but can demand a considerable amount of time and personnel to generate and maintain. Learners are repeatedly exposed to outdated or incorrect information when existing knowledge tests become outdated as source information is modified or removed. In high-risk professions, such as medicine or aviation, it is imperative that learners have access to the most up-to-date corpus of documents and study materials.

**Figure 1:**
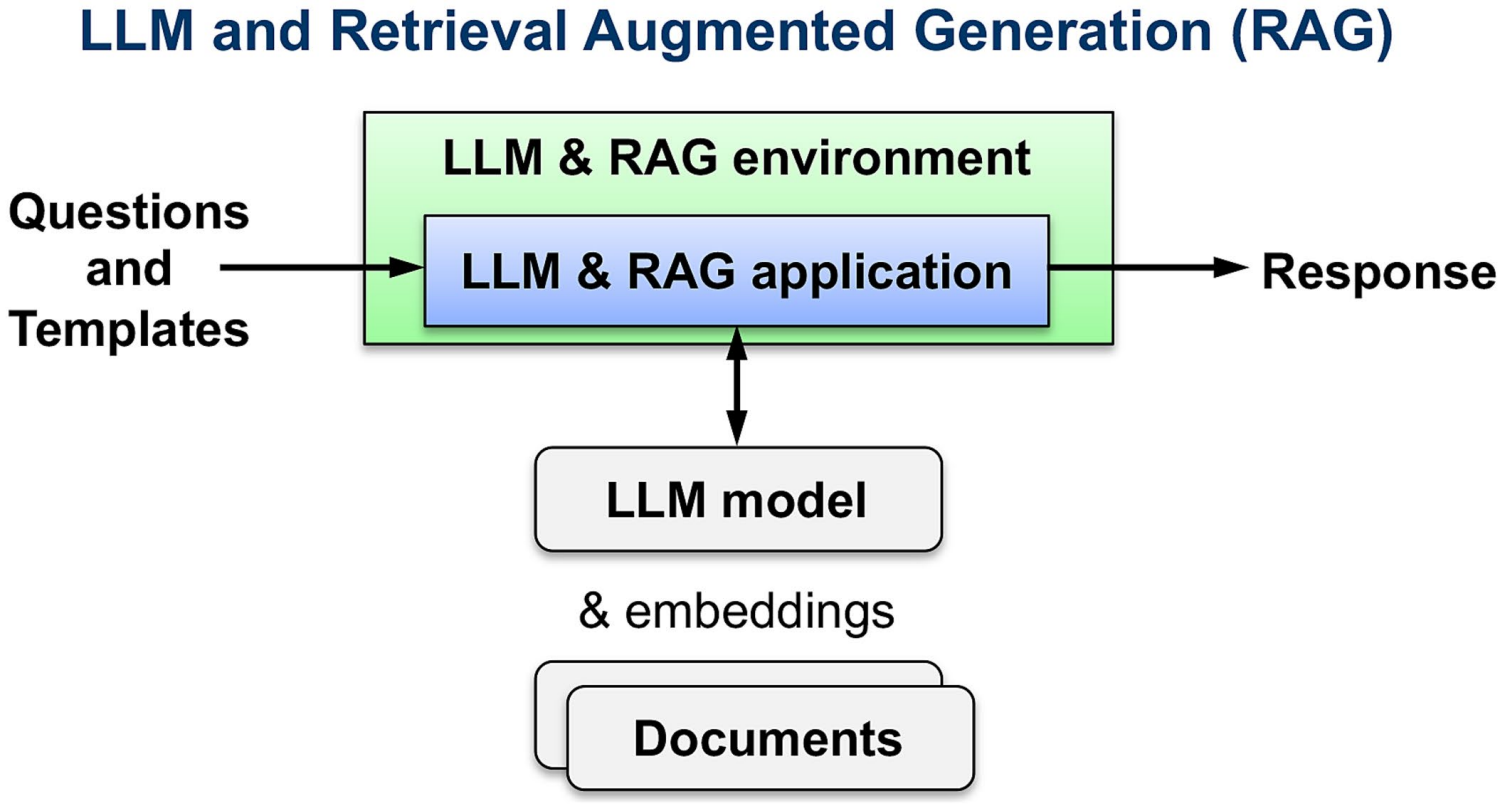
Large language models (LLM) and Retrieval-Augmented Generation (RAG) overview.

Recent development of Large Language Models (LLMs) combined with Retrieval-Augmented Generation (RAG) of documents and information not included in the LLM training data provides a framework of technology solutions to address aspects of these education challenges. Multiple documents can be embedded into one or more embedded databases or vector stores. LLM RAG can be used to query the knowledge base for specific questions; this enables rapid lookup of information across multiple large documents (Figure 1). LLM RAG implementation performs very well on question-answering (QA), fact verification, and attribution tasks while hallucinating less than other methods (Wu et al., 2024; Lewis et al., 2020). However, current LLM RAG capabilities fall short of fully utilizing the context of a document; LLM RAG is susceptible to what is known as the *lost-in-the-middle* challenge, where the LLM struggles to fully utilize information hidden within a long context (Liu et al., 2023; Xu et al., 2023). If implemented for knowledge-intensive professions with current methods, critical information may be lost or overlooked.

To evaluate LLM RAG for enhancing and facilitating education on complex, jargon-dense, closed-library documents, the Artificial Intelligence for Knowledge Intensive Tasks (AIKIT) system was developed. To provide portability, AIKIT has been containerized in both Singularity (Kurtzer et al., 2017) and Docker (Merkel, 2024) containers and a Conda environment. AIKIT includes a Ruby on Rails web user interface. AIKIT is being released as open source at https://github.com/mit-ll/AIKIT.

## 2 Materials and methods

### 2.1 Document as a Dictionary—DaaDy

To solve the problem of incomplete text utilization for LLM RAG on large documents, Document as a Dictionary—DaaDy was developed. DaaDy is a framework in which LLM RAG can be systematically completed on each section/subsection/sentence of a document. This method takes structured documents (documents with headings, sections, and/or subsections), parses them, and stores the entire document as a series of nested dictionaries where the highest-level key is a heading/section/subsection title, and the lowest-level value is an individual sentence from the document. This is implemented with two Python tools, one for parsing a document into a DaaDy (afman_parser.py) and another to consolidate multiple dictionaries (daady_consolidator.py). Storing metadata in this dictionary framework enables added functionality for source attribution of LLM RAG responses. The DaaDy framework allows the prompt to be queried against all sections of a document by loading each section/subsection/sentence into the retriever, individually; context length remains short enough to achieve full utilization in LLM RAG. A dataset of regulatory and procedural documents from the United States Air Force were utilized in this study, including documents containing various types of flying rules and regulations. In this dataset all documents have a standard format for the title, header/footer, table of contents, and paragraph headings. All Air Force Instructions (AFIs) and Manuals (AFMANs) use the same numerical paragraph heading structure. Top-level headings begin at 1.1, second-level headings at 1.1.1, and so on. The DaaDy tool cleans and consolidates sentences from all paragraph levels and produces two DaaDys, a Section DaaDy and a Sentence DaaDy. The Section DaaDy cleaned and stored text into all applicable sections—for example, if a sub-section started with the header “1.1.3,” the text within that subsection would be cleaned and stored in both the “1.1” section and “1.1.3” sentence DaaDy, effectively turning a structured document into groups of contextually similar paragraphs of varying lengths. The Sentence DaaDy stored each sentence individually in the lowest-level dictionary. The parser used in this study uses regular expressions to recursively parse the document. It was designed specifically for AFI and AFMAN formats and is programmed to parse expected headings from the table of contents and clean footers from the text. With small updates to the regular expressions (for table of contents, footer, and paragraph header), afman_parser.py could be easily tailored to any document with sequential paragraph headers.

### 2.2 Structured Question Answer Dictionary—SQAD

To combine LLM RAG with DaaDy, the method called Structured Question Answer Dictionary, or SQAD was developed. SQAD is able to generate new material for knowledge tests, with each item made up of a question (Q), an answer (A), and a section or paragraph reference (R), henceforth referred to as QAR. SQAD can also be used to locate context and assess the validity of existing QARs in knowledge tests after document revisions in the knowledge base. The expedient LLM RAG assessment of current QARs and generation of new QARs on updates to the knowledge base can provide benefits to instructors and learners in knowledge-intensive professions.

### 2.3 Containerized AI for knowledge intensive tasks (AIKIT)

**Figure 2:**
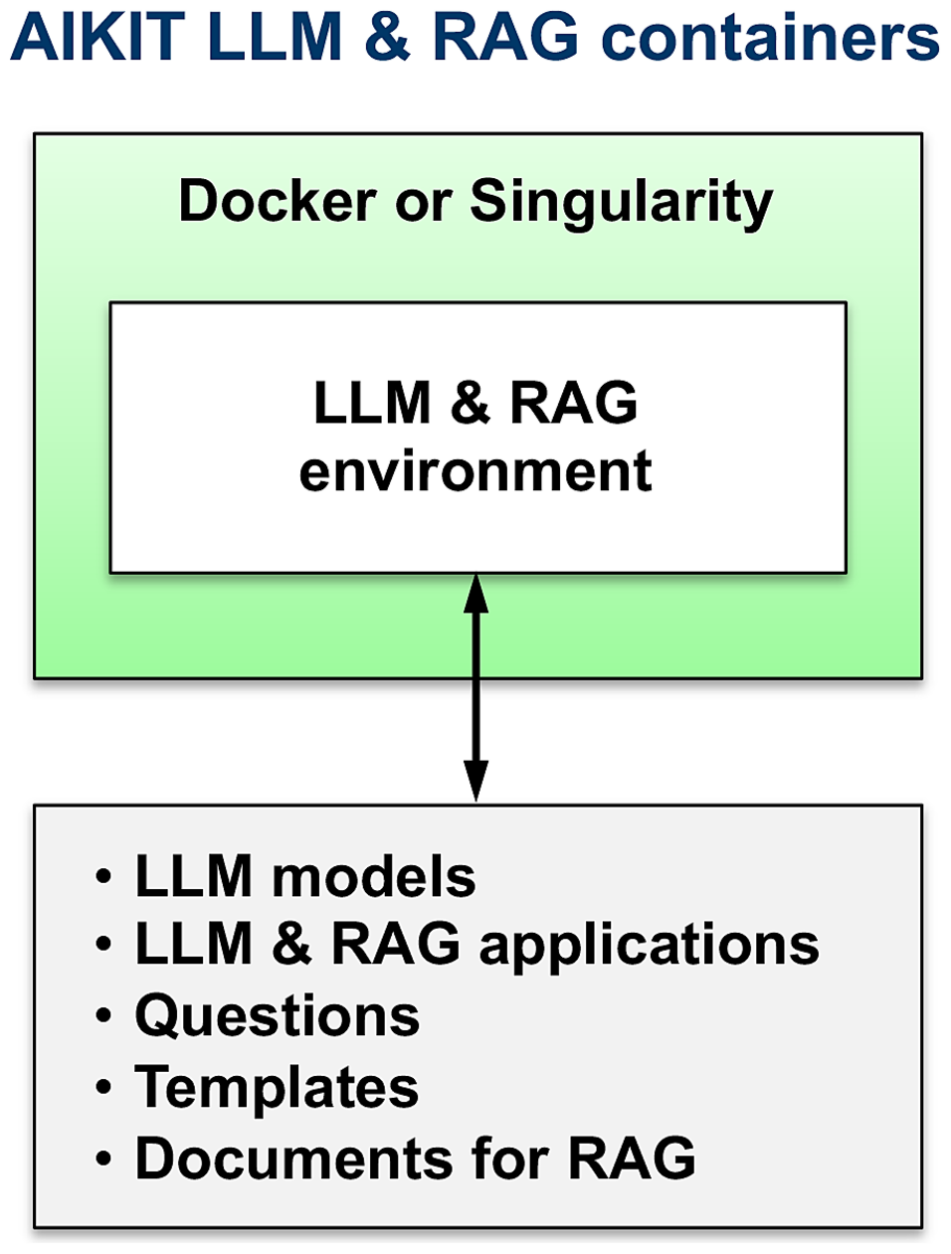
Docker and singularity containerized AIKIT.

**Figure 3:**
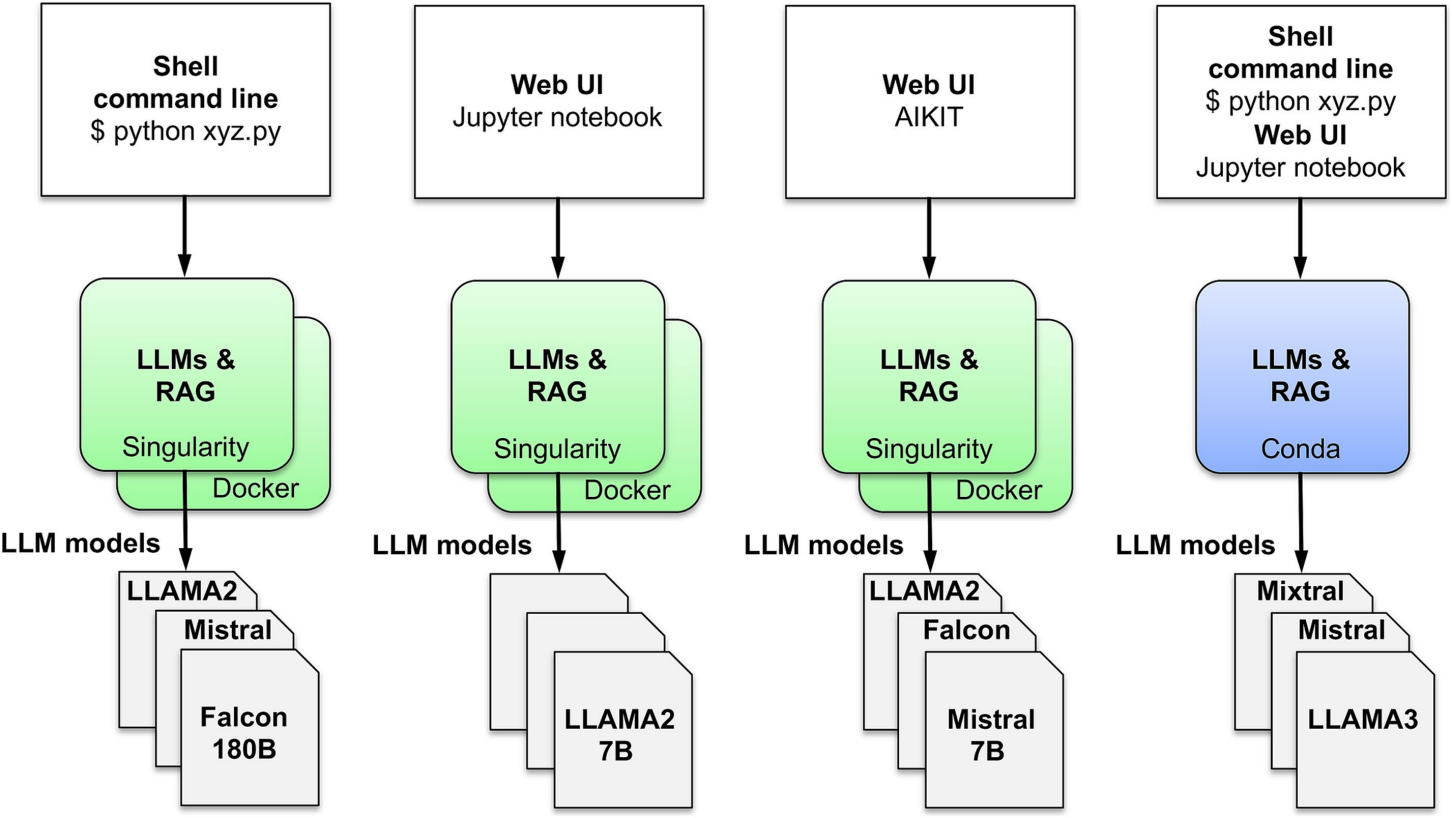
AIKIT command line and web interfaces.

To easily enable hosting on multiple platforms, AIKIT was packaged into Singularity (Kurtzer et al., 2017) and Docker (Merkel, 2024) containers (Figure 2). AIKIT is also packaged in a Conda environment (Figure 3).

### 2.4 Large language models and retrieval-augmented generation

AIKIT is not dependent upon any specific LLM. The LLM models Mistral-7B-instruct-v0.2 (2023) and Mixtral-8x7B-Instruct-v0.1 (2024) models from Mistral AI, and other models have been used with AIKIT. LLM RAG was implemented in Python (Python programming language, 1991) (v3) with LangChain (2022), vector stores (embedding databases) FAISS (2017), and Chroma (2022), and HuggingFace embeddings model sentence-transformers (all-mpnet-base-v2, 2021). The LangChain PyPDFLoader (2021) was used for parsing Adobe portable document format (PDF) documents. Paired Python tools were developed to create vector stores (docs_to_vs.py) and LLM RAG queries (llm_rag_query.py). These two Python tools accept JavaScript Object Notation (JSON) parameter files for input.

### 2.5 Web interface

AIKIT user interface was developed in RubyOnRails (2004) (v7.0.1) and Ruby (1995) (v3.0.3). The SQLite3 database was used for development, but AIKIT will work with any Rails supported database. The AIKIT user interface invokes the Python tools docs_to_vs.py and llm_rag_query.py to create vector stores and query LLM RAG targets, respectively.

### 2.6 Multi-GPU enabled systems

Singularity container and nvccli options were utilized to parallelize across all of the available GPUs on the hosting platform.

When running with --nvccli, by default SingularityCE will expose all GPUs on the host inside the container. This mirrors the functionality of the legacy GPU support for the most common use-case. Setting the SINGULARITY_CUDA_VISIBLE_DEVICES environment variable before running a container is still supported, to control which GPUs are used by CUDA programs that honor CUDA_VISIBLE_DEVICES.

However, more powerful GPU isolation is possible using the --contain flag and NVIDIA_VISIBLE_DEVICES environment variable. This controls which GPU devices are bound into the /dev tree in the container. For example, to pass only the first GPU into a container running on a system with multiple GPUs, one would export the following variable values as shown below to achieve this:

export NVIDIA_VISIBLE_DEVICES = 0.

export SINGULARITY_CUDA_VISIBLE_DEVICES = 0.

The Singularity contain and nvccli options were used with GNU Parallel (GNU, 1983). A master shell script was created for each GPU with a text file containing the commands to run.

### 2.7 Prototyping environment

AIKIT development and prototyping efforts were performed on both x86 and ARM-based architectures. The x86 system had two Intel Xeon Gold 6258R CPUs, 256GB RAM, and an NVIDIA RTX A6000 GPU. The ARM-based system had an Apple M2 known as a system on a chip which serves as both a CPU and a GPU, 8GB RAM, and a 256GB solid state hard drive.

### 2.8 HPC system implementation (2-NVIDIA-V100)

The MIT Lincoln Laboratory Tx-Green system (2-NVIDIA-V100) (TX-Green, 2024) was used as the high performance computing system for our pipeline prototype development. The GPU systems have Intel Xeon PHI 7210 64C 2.5 GHz CPU with 40 cores, 377 GB RAM, Intel Omni-Path with 2 NVIDIA Tesla V100 GPUs. LLMapReduce was used to submit jobs to the SLURM queue (Reuther et al., 2018).

## 3 Results

### 3.1 Document as a Dictionary—DaaDy

**Figure 4:**
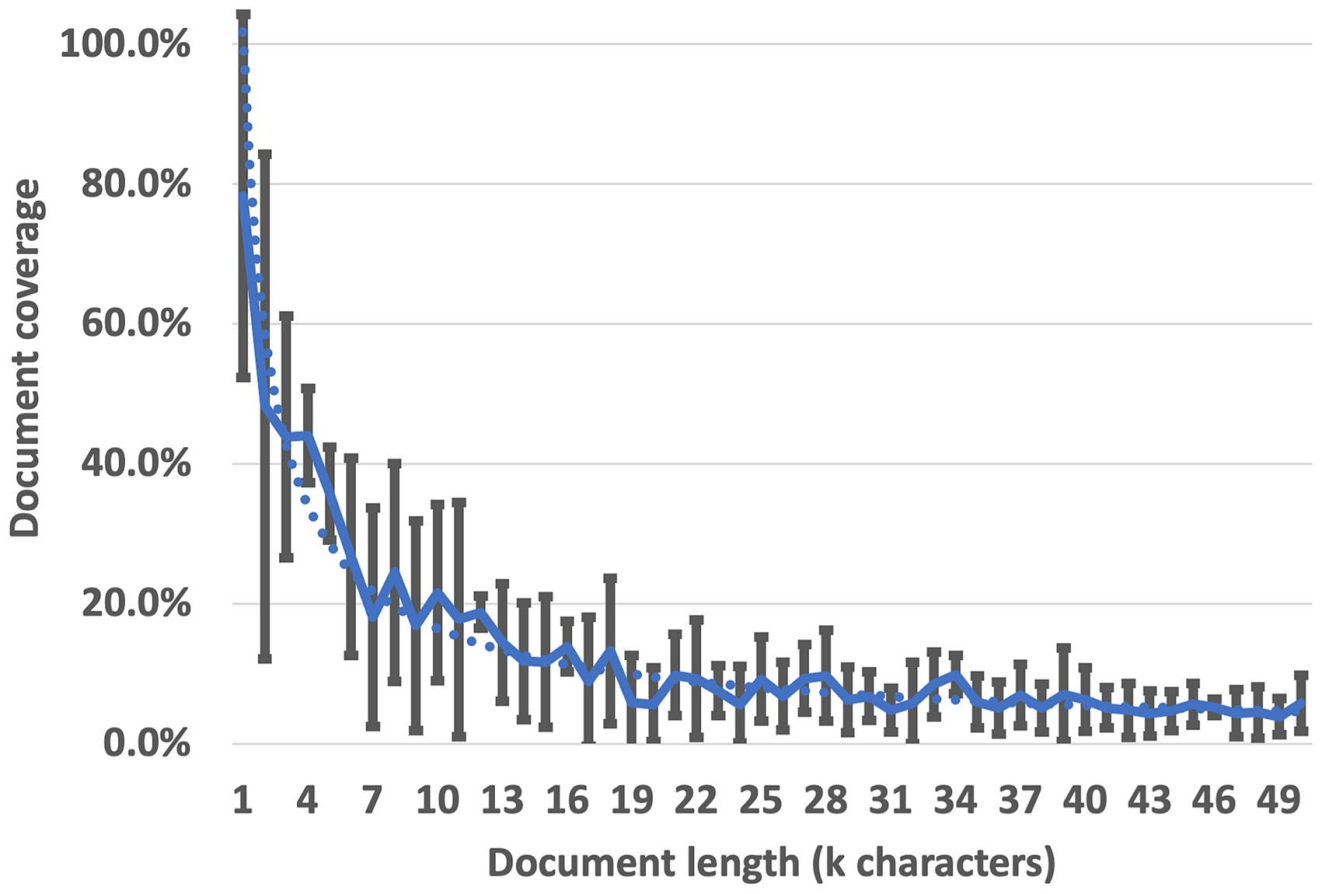
Document coverage by LLM RAG generated questions.

Figure 4 shows that while the specific oscillations differ between documents and individual runs, a strong trend of decreasing context utilization is consistent across all cases during 300 attempts. In no case did the LLM RAG utilize more than 25 percent of the context when the document was longer than 18,000 characters. On average, across all 6 context bases, less than 20 percent of the context was utilized when documents were longer than 10,000 characters and less than 10 percent of the context was utilized when documents were longer than 20,000 characters; our data suggests a full-utilization maximum of between 1,000 and 2,000 characters. While research seeking to decrease the magnitude of this effect continues, instructors and learners who intend to use LLM RAG to generate training material currently lack the capability to do so effectively on long documents without losing critical information. The DaaDy framework was developed to enable QAR generation coverage of the document sections individually and ensure all desired content is utilized.

### 3.2 Test questions generation

**Figure 5:**
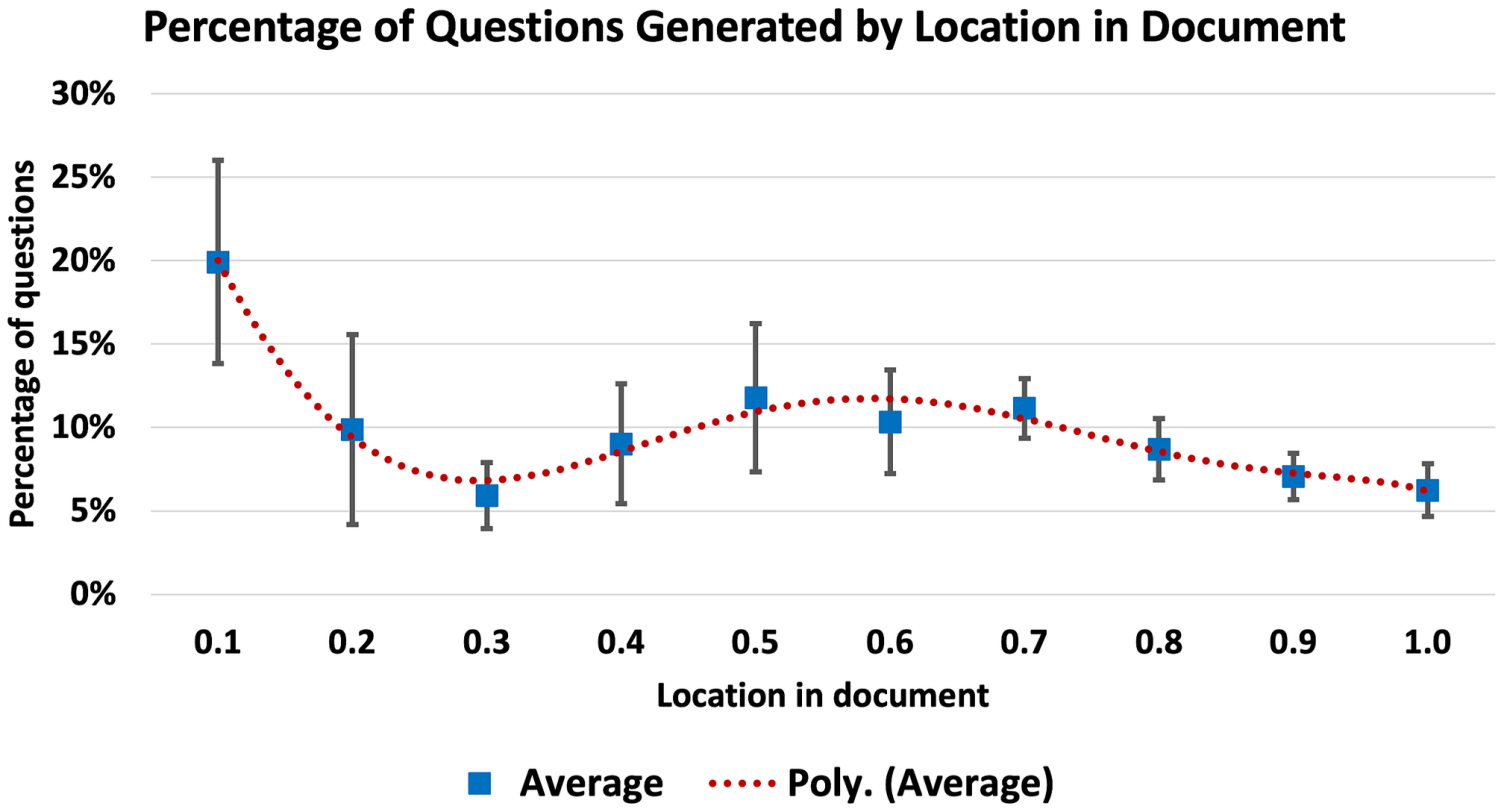
Context utilization in varying document lengths.

Question, Answer, Reference (QAR) groups were generated on selected documents with LLM RAG. The goal was to comprehensively utilize the material in the selected documents from which a subset of useful, accurate, and well-phrased questions could be selected. A prompt was given for the LLM to generate a QAR for each sentence in the document which was longer than five words (see Appendix A for final prompts used in this research). Initially, this prompt was implemented on the document in its entirety, and a significant amount of context was unrepresented in the questions generated. Very high content coverage was observed for documents less than 1,000 characters in length, measured by assessing the number of QARs output divided by the number of sentences in the document which were greater than five words long (a result of 1.0 was assessed as full context utilization). To study this effect further, the prompt was tested on documents of varying lengths in order to assess where information was being utilized and lost; six documents were used in total (Figure 4). The prompt was implemented and from the output, the location of each reference was derived as a percentage of the full document length. A noticeable bias of content from beginning of the document was noted (Figure 5) with 5 of 6 documents showing between 17 and 26 percent of the questions generated originating from the first 10 percent of the document (a single outlier at 9% was observed). In the 6 documents examined, underrepresented QAR coverage of documents was observed for locations at 30, 90, and 100% (Figure 5).

To mitigate the lost-in-the-middle effect, DaaDy was created. DaaDy takes a document as the input and separates the document into a series of nested dictionaries containing sections, subsections, and sentences. While future users could customize the base-level of DaaDy to their needs, our testing used the sentence as the lowest level value in the dictionary. SQAD calls the prompt separately on each desired section of the dictionary, creating a QAR for each sentence in the document. This also permits the storage of metadata about each sentence in the document, which by alleviating the LLM from the responsibility of correctly interpreting and storing data from the text, allows the user to store and retrieve sentence-level metadata with perfect recall.

Unsurprisingly, implementation of the prompt on sentence-level DaaDy data resulted in a perfect score for context utilization: for a 105,000 character-long document, 910 QARs were produced in approximately 24 min and 30 s, resulting in a per-question QAR time of 1.62 s on an ARM-based system. The Chief Instructor Pilot from a USAF Fighter Squadron was asked to review the QARs and check them against the source document. This expert was asked to grade the utility, accuracy, and phrasing for each QAR. If the QAR needed no amendment to be useful, accurate, or well-phrased, the expert was instructed to provide no remarks for that attribute. For anything less than this criterion, the expert was asked to write a statement explaining what exactly was suboptimal for each attribute. Most questions (354 out of 477) received no remarks for utility, accuracy, and phrasing. The remaining 123 QARs were considered anomalous for one or more of the attributes. The expert’s notes were analyzed to understand, categorize, and describe these issues. There were seven main categories of anomalous QARs which emerged from the data (see Appendix B for definitions and examples): unable to answer, repetitive QA, unnecessary justification, missing context (lists), non-sequitur, misleading QA, and acronym hallucination. For both SQAD question generation and evaluation, significant degradation in LLM RAG performance was observed when niche acronyms were used or phrases were used outside of their normal context.

### 3.3 Test questions evaluation

**Figure 6:**
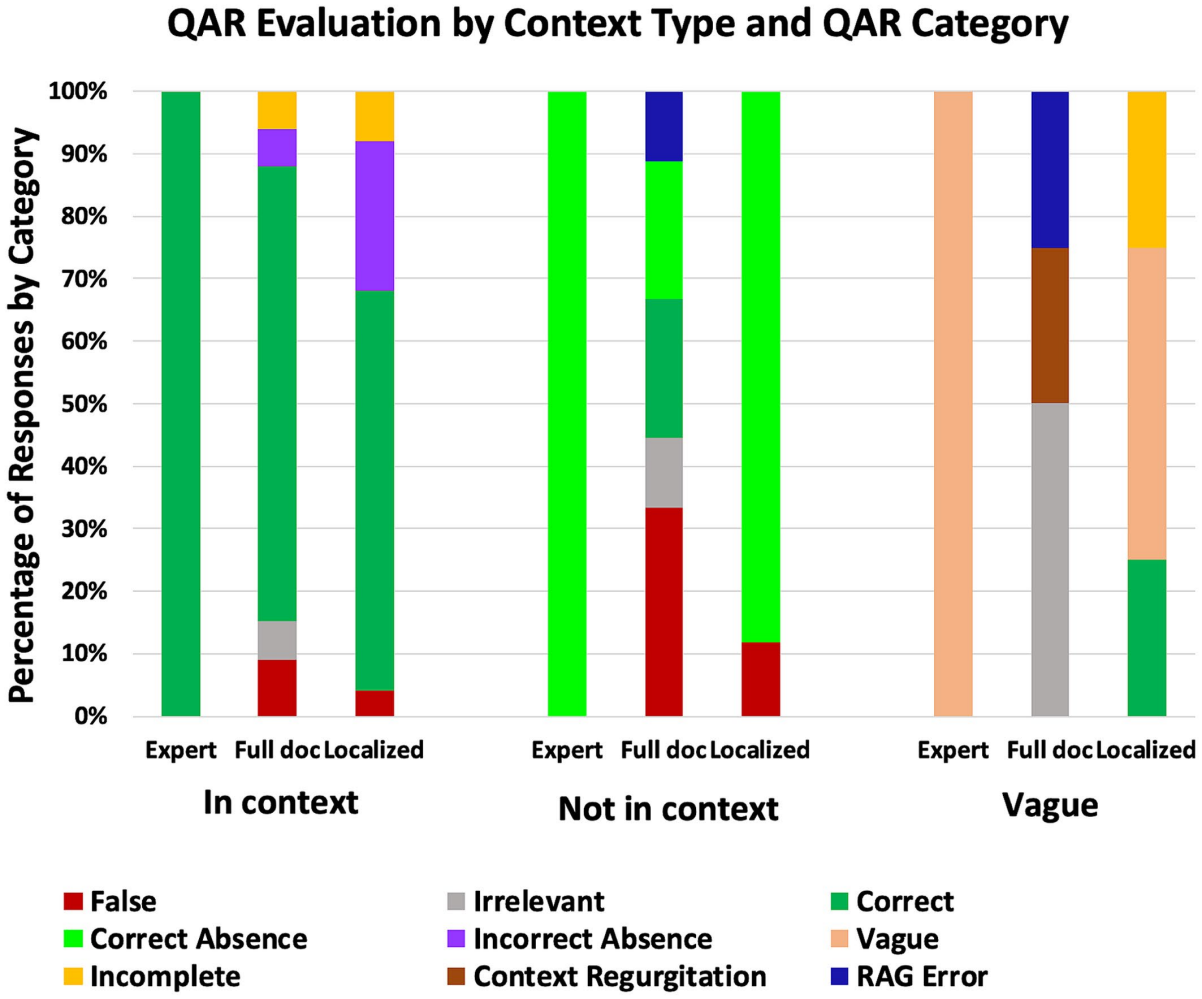
Context-based question evaluation versus expert assessment.

Outdated test questions based on updated publications were evaluated with LLM RAG on documents via SQAD to identify whether the question was (1) still supported by the knowledge base, (2) in need of revision, or (3) if relevant content had been removed. Two question-evaluation trials were run. First, each question in the test was posed using the entire source publication as the context. Second, the same queries were made using only the localized context from the DaaDy as search context. The results of these methods were compared against an expert’s assessment of the test questions. The expert compared each QAR against the current source publication and given paragraph reference from the source document. The answer was categorized into one of three bins: (1) correct answer contained in specified reference context, (2) correct answer not contained in specified reference context, (3) question verbiage so vague that a specific, correct answer could not be reasonably determined. Once this gold standard was established, the expert graded the answers generated in each of the two trial methods and was again asked to create and categorize each response. If the answer was contained in the specific reference context and the LLM RAG query produced the correct answer, that was categorized as a correct response; the opposite would be a false response. If the correct answer was not contained in the specific reference context, the LLM RAG query could produce either a correct absence or an incorrect absence. Five other distinct categories emerged from the data (see Appendix C for definitions): vague response, irrelevant response, incomplete response, RAG error, and context regurgitation responses. The results of these two trials are summarized in Figure 6.

### 3.4 AIKIT user interface

A Ruby on Rails web interface was developed for AIKIT. The AIKIT UI includes a user interface enabling access to documents, document queries (LLM RAG), tests, and test results. LLM model queries and LangChain (2022) chaining of questions is also included. The instructor interface is also included with access to test questions and answers, and evaluation of test questions.

### 3.5 Documents query

Querying knowledge base documents is implemented in AIKIT as standard RAG embedding of documents with a LLM. Queries can be run via command line, Jupyter notebook, or AIKIT web interface (Figure 3). The AIKIT web interface database retains query results.

## 4 Discussion

### 4.1 SQAD

The DaaDy framework combined with SQAD for QAR generation resulted in 100% content utilization in large documents, a significant improvement over current methods. As the quality of a question stems directly from the utility of the source context and the studied documents lack an accepted metric for relative or absolute sentence utility, no quantitative data was generated from this study to determine whether the question quality using DaaDy/SQAD was superior or inferior than single-prompt LLM RAG. While quantitative observations were not produced, there were a number of relevant qualitative assessments made based on the observation of SQAD QAR-generation. By using a single sentence as the context provided to the LLM RAG, a significant portion of context/background knowledge was removed from the LLM RAG, which may have caused at least four of the seven categories of anomalous QAR generation (unable to answer, repetitive QA, missing context-lists, non-sequitur, and possibly, misleading). Rudimentary trials (data not shown) showed that, generally, when context length was kept to less than 1,000 characters, the full context was utilized for QAR generation. Thus, we hypothesize that if the SQAD method instead of passing a sentence, passed 1,000 or less characters that group together coherent sentences, paragraphs, or sections within the DaaDy, the generation of anomalous QARs would decrease while maximizing context utilization.

In the area of SQAD QAR evaluation, three scenarios were studied. When the answer was contained in the provided context, LLM RAG of the full document performed better at QA than the localized context (72.7% vs. 64%), see Figure 6. Additionally, QA on the localized context reported incorrect absences significantly more than when queried against the full document (24% vs. 6.1%) (Figure 6). When the answer was not contained in the provided context, RAG of the full document produced significantly more false (33.3% vs. 11.8%) and irrelevant (11.1% vs. 0%) responses than querying only the localized context (Figure 6). We also observe that the full-document LLM RAG malfunctioned more than the localized-context LLM RAG, producing RAG errors (11.1%) whereas the localized-context RAG produced none (Figure 6). While the study of answering poorly-phrased questions lacks significant benefit, it is interesting to note that the full-document query produced irrelevant responses (50%), RAG errors (25%), and context regurgitation (25%) responses, while the localized-context query either accurately recognized the vagueness and reported that insufficient context was provided to answer the question (50%), provided a correct but incomplete response (25%), or stated that the answer was not contained in the context (25%) (Figure 6). From this data, we draw the conclusion that an increased quantity of background information permits higher certainty on QA when the answer is contained explicitly in the context. However, when the answer is not contained in the provided context, the presence of extraneous material produces undesirable (irrelevant and false) responses as well as text-generation malfunctions (RAG errors and context regurgitation). Using localized context in these cases produce a more desirable and transparent result.

For the purpose of SQAD QAR evaluation, there is a key difference between the definitions of responses which were deemed “false” or “hallucinated.” Answers were categorized as “hallucinated” when the answer included information which was not found in the source document. In this study, this was almost always the result of the LLM attempting to spell out an acronym which was not defined in the source document. This could be ameliorated in the future by including an acronym list or adding instructions to the prompt to avoid spelling out acronyms. Answers were categorized as “false” when the answer only included information which was found in the source document but the answer to the question was incorrect. This usually occurred when the answer to a question required synthesizing information found in multiple, separated sentences in a document or multiple documents.

The use of DaaDy and SQAD creates a framework where LLM RAG behavior is more predictable and the context utilized can be known with high fidelity. Due to this increase in both transparency and predictability, we assert that LLM RAG can be implemented as a tool to improve human efficiency in knowledge-intensive professions. The importance of expert supervision and quality assurance cannot be understated. LLM RAG enhanced with SQAD and DaaDy can increase efficiency and comprehensiveness are still susceptible to the aforementioned anomalies observed in text generation. Thus, it is absolutely critical that these methods be utilized with appropriate levels of supervision and a framework for quality assurance, else the enormous increase inefficiency could turn into a rapid spread of false information (Fernando, 2023).

### 4.2 AIKIT user interface

Access to LLMs currently is via graphical user interfaces or frequently by developing small Python programs. New interfaces providing LLM RAG capabilities are being rapidly developed. Getting the technical details connected properly is a barrier for many projects to easily access LLM RAG capabilities. The two Python tools docs_to_vs.py and llm_rag_query.py provide configurable access to creating LLM RAG embedded documents and querying them. The Ruby on Rails AIKIT web interface profiles configurable creation and querying of documents in LLM RAG knowledge bases. AIKIT provides web viewing and downloading of knowledge base documents. AIKIT also includes support of test-taking with feedback on test questions to instructors. LLM RAG queries and responses and test question responses for learners are retained in the AIKIT database.

AIKIT tools can be by command line interfaces, via Jupyter notebook, or Rails interface. The utility of AIKIT has been increased to include multiple document types including Microsoft Word, Excel, PowerPoint, text, voice, text within images, and automatically generated description of content within images for LLM RAG queries. To increase user friendliness, multiple different levels of user interface capabilities were developed to enable alignment of user needs with desired AIKIT capabilities. Multiple unrelated research efforts are currently ongoing applying AIKIT to multimodal LLM RAG applications highly leveraging the multiple document types supported.

### 4.3 Recommendations for knowledge base management

This study focused on a document corpus which had an associated framework for QA. Fields which lack this formal infrastructure but require professionals to learn and commit vast amounts of information to memory may want to consider creating this QA framework. SQAD will help accelerate the process of turning documents into QARs and can minimize time required for manual updates. Both should be supervised by an expert before QARs are put into use. Finally, this study focused only on documents that were highly structured. While parsing structured documents is very simple, this structure is not required to use these methods; the parser’s code could be updated easily to assign an index number to each sentence and use that index number as a reference in absence of a paragraph header. Creating sections which provide logical context, such as the “Section DaaDy” does for structured documents, will be a challenge for managing knowledge stored in less structured documents, as the user’s available chunking mechanisms are punctuation and white space characters.

Throughout this research there were numerous roadblocks that, if avoided, will significantly improve or simplify the process by which LLM RAG can be wielded to assist in knowledge-intensive professions. Well-structured documents can make the parsing from text to DaaDy expedient and easy. First, maintaining the master copy of each document in the corpus in a purely text form (void of headers, footers, page numbers, and other formatting characters) will significantly ease the burden on coding and debugging automatic parsers. Using word-processing software that encodes the document structure in text form that can be parsed using regular expressions (Rossum, 1999) will simplify the process by which the knowledge can be accessed using LLM RAG. Finally, for professions that generate and maintain QARs, avoiding the following will allow straightforward usage of LLM RAGs for test evaluation: (1) avoid asking vague or open-ended questions, (2) avoid using different verbiage in the question than in the context (e.g., “night” versus “between sunset and sunrise”), (3) avoid referencing the publication title in the question unless that data is included in the prompt.

## 5 Future work

The results of this research showed that while there is currently an upper limit to the length of context that can be fully utilized effectively by LLM RAG, there is also a minimum length at which the context is so isolated that its utility decreases to the point of difficulty and inconvenience for the user. In future iterations of SQAD, research should be pursued to determine the optimal context length and chunk size to maximize effective context utilization. Further inquiry into whether there is any relationship between chunk size and presence (and type) of anomalous response would be a worthwhile contribution. Once these parameters are defined, LLM RAG can be optimized for question generation and evaluation. Improvements to LLM RAG should provide sentence context metadata aligned with the document’s structure.

The AIKIT UI, due to its fully offline implementation, has the potential to transition to secure systems. The ability to use AI in querying and updating a vast knowledge base while keeping one’s data and documents secure has enormous potential in many fields with highly-restrictive security requirements.

## 6 Conclusion

While the capability of LLMs to produce human-like, accurate, and attributable responses has improved significantly in recent years, LLM RAG utilization of text in long documents is an area in need of improvements; these deficiencies render LLM RAG unsuitable as a tool for professions which require accountable and full utilization of the profession’s knowledge base. The document organization framework, DaaDy, and the querying method, SQAD, presented in this paper significantly improve the utilization rate of LLM RAG over long documents and provide transparency for QA tasks. By utilizing SQAD and DaaDy, human expertise and intuition can be enhanced by expedient context-querying and content generation.

Additionally, the AIKIT prototype is a fully-containerized, offline solution which can be easily deployed on laptops, workstations, high-performance computing (HPC) clusters, and cloud solutions. AIKIT can thus provide easy-to-use LLM RAG to a wide audience. AIKIT runs on any platform—from a system on a chip (SOC) to HPC or cloud infrastructure. AIKIT is being released as open source at https://github.com/mit-ll/AIKIT. Please contact the authors with questions, requests, or feedback.

## Data Availability

The raw data supporting the conclusions of this article will be made available by the authors, without undue reservation.
