# Supplementary material for: Large language models for closed-library multi-document query, test generation, and evaluation
Source: Front Artif Intell. 2025 Aug 6;8:1592013. doi: 10.3389/frai.2025.1592013 (PMC12364804; doi:10.3389/frai.2025.1592013)
Supplement: Supplementary file 1 [file Data_Sheet_1.docx]

Appendix A: Prompts for SQAD Generation and Evaluation

Prompt for SQAD QAR Evaluation ^1^:

prompt_input = """Answer the following question as concisely as possible

based only on the provided context. If you are not sure of the answer,

your answer should be "Insufficient context provided."

Keep your answer short and to the point. Avoid using the words "the context"

in your answer.

<context>

{context}

</context>

Question: {input}"""

Prompt for SQAD QAR Generation:

The input prompt used for this research was built off of previous research from Wu et al. ^2^

prompt_input = """Answer the following question based only on the provided context:

<context>

{context}

</context>

Question: {input}""")

input = """You are given context that contains information. Generate a

question and answer pair that tests the reader's understanding of the

information contained within the prompt. Each question should provide

sufficient context so that the question does not require the context to be

fully understood. Do not reference the word 'context' in the question or

answer. The question should have a straightforward answer that can be easily

verified with the context. In addition to the question, provide the answer that

is succinctly taken from the prompt.

Provide the question and answer in the following format:

Question: Provide the question here.

Answer: Provide the Answer here. Make the answer as concise as possible."""

Appendix B: SQAD Generation - Anomalous QAR Category

Definitions and Examples

1. Unable to Answer - the QAR produced included a question that the context did not answer, to which it correctly answered that the answer was not available in the context provided. Example:

• Question: Under what circumstances are runway operations automatically suspended?

•. Answer: Runway operations are automatically suspended when not specified in the context. The context does not provide information on the specific reasons for suspension.

1. Repetitive QA - the QAR produced included a question and answer pair where the answer was a part of the question, resulting in either a confusing question or an obvious answer. Example:

• Question: In what situation might you need to evacuate an airport runway in an emergency?

•. Answer: In an emergency runway evacuation.

1. Unnecessary Justification - the QAR produced included a, generally obvious, justification after the answer despite the fact that it was prompted to avoid doing so and that the context was very short. Example:

• Question: Who or what must approve cable certifications?

• Answer: The 20 OG/CC must approve cable certifications. Answer derived from the prompt: Cable certifications must be approved by the 20 OG/CC.

• Context: Cable certifications must be approved by the 20 OG/CC.

1. Missing Context (Lists) - the LLM RAG was prompted on each sentence, resulting in the complete loss of context when the sentence was a part of a list of items or actions. When sub-items in a list were taken out of context, the result was inaccurate and confusing information which could be seriously detrimental to the learner if not caught. Example:

• Question: Under what circumstances are pop patterns allowed in this situation?

• Answer: Pop patterns are allowed only if required for syllabus sorties.

• Localized Context: No pop patterns unless required for syllabus sorties.

• List context:

- 2.4.2. [Bird Watch Condition] Moderate Low approaches and chase aircraft limited to events that could not be accomplished at other airfields and are required for training or checkrides. No formation approaches.
- 2.4.2.1. Low Altitude Structure. 1,000 feet AGL minimum, except as required for weapons delivery (defined as within 10 NM of target). If low altitude currency is required for contingency operations or training requirements, minimize time in low altitude structure below 1,000 feet AGL.
- 2.4.2.2. Poinsett Range. 1,000 feet AGL minimum, except as required for weapons delivery (defined as base position for the curve pattern). **No pop patterns unless required for syllabus sorties.**

• Explanation: The question uses the verbiage in this situation rather than during a Bird Watch Moderate condition which lacks the critical connection between cause and effect for this context.

1. Non-Sequitur - when context was very short and non-descriptive, LLM RAG generated what the expert categorized as accurate but useless QAR. Example:

• Question: In what domain does the term Airfield Lighting Systems belong?

• Answer: Airfield Lighting Systems belong to the domain of aviation or airfield infrastructure.

1. Misleading - in an effort to be concise, LLM RAG would produce a QAR that was misleading. Example:

• Question: On which Mondays are both runways open for use?

• Answer: Both runways are open on Mondays that are not the 1st, 2nd, 3rd, or

4th Monday of the month.

• Context: Runway 04L/22R will close every 1st/3rd Monday and Runway 04R/22L

will close every 2nd/4th Monday of the month from 0700L to 1100L for preventative maintenance (PM).

• Explanation: The QAR produced indicates that both runways are closed on the 1st, 2nd, 3rd, and 4^th^ Monday of the month; in reality, only one runway is closed between 0700L and 1100L.

1. Acronym Hallucination - in many cases where an acronym was used, LLM RAG hallucinated the full title and provided the incorrect entity in the question and/or the answer. Example:

• Acronym: SOF

• Correct Entity: Supervisor of Flying

• Hallucinated Entity: Senior Operations Force commander

Note: While there was insufficient data to report this formally as a finding, initial results of attempting queries on larger chunks (fewer than 1000 characters) of data appeared to significantly improve many of these anomalies, including "unable to answer," "missing context," and "non-sequitur."

Appendix C: SQAD Evaluation - Response Category Definitions

1. False Response - the response provided was an incorrect answer to the question.

2. Irrelevant Response - the response provided was not relevant to the question asked.

3. Correct Response - the response provided was assessed as correct by the expert.

4. Correct Absence - the response accurately reported that the answer to the question

was not contained within the provided context.

5. Incorrect Absence (false negative) - the response inaccurately reported that the answer

to the question was not contained within the provided context, even though it was.

6. Vague Response - the response accurately reported that there was no specific answer

to the question asked.

7. Incomplete response - the response provided was true but was missing some critical

information from the same context. Example:

8. RAG Error - the response provided was obviously cut off mid word, acronym, or

sentence.

9. Content Regurgitation - the response provided was an excerpt or series of excerpts

copied exactly from the context.

**References**

1. RAG. Mistral AI Basic Retrieval-Augmented Generation (RAG). Accessed July 14, 2024, <https://docs.mistral.ai/guides/rag/>

2. Wu K, Wu E, Cassasola A, et al. How well do LLMs cite relevant medical references? An evaluation framework and analyses. *arXiv*. 2024;doi:<https://doi.org/10.48550/arXiv.2402.02008>
